# Situated Learning and Transnational Labor Migration: The Case of Canada’s Seasonal Agricultural Worker Program

**DOI:** 10.1177/07417136221095480

**Published:** 2022-04-18

**Authors:** J. Adam Perry

**Affiliations:** Department of Adult Education, St. Francis Xavier University, Antigonish, Nova Scotia, Canada

**Keywords:** situated learning, migrant workers, agriculture, workplace learning, immigration

## Abstract

Grounded in an analysis of interviews with migrant farm workers in Canada, this article explores how learning in the everyday contexts of temporary transnational labor migration is implicated in both migrant identity formation and the social reproduction of an established and growing labor migration regime. The article focuses on thinking through how workers negotiate the intergenerational workplace tensions that permeate life in Canada's Seasonal Agricultural Worker Program. The findings suggest that through their sustained participation in the everyday social practices that develop through dormitory-living, transnational laborers learn to *become* migrant workers. This formation of migrant worker identities in turn contributes to the reproduction of the social relations that support the ongoing practice of circulatory labor migration in the Canadian agricultural industry.

## Introduction

The experience of international migration is intimately linked with that of work and lifelong learning (English & Mayo, 2019). There is a growing literature from the field of adult education that examines the dimensions of this interlocking relationship, as is evident from recent special issues (Guo & Lange, 2015; Guo & Maitra, 2019; Morrice et al., 2017), edited volumes (Guo, 2013; Hoggan & Hoggan-Kloubert, 2022), and numerous book chapters (Guo, 2018; Morrice, 2018; Shan, 2018; Shan & Guo, 2021). The vast majority of this research is focused on thinking through the relationship between adult learning and the socio-economic inclusion of immigrants and refugees who permanently relocate across international borders, normally from low-income to high-income countries. Much of this research is grounded in qualitative empirical analysis and has made sophisticated and important contributions to the fields of adult education and lifelong learning as they intersect with issues related to international migration. Canadian adult educators in particular have made important advances in excavating the role that lifelong learning regimes may play in the systemic racialization and exclusion of immigrants and refugees from host societies, particularly as concerns the experiences of women migrants (Maitra, 2015; Ng & Shan, 2013). However, the experiences of migrant workers, who in 2017 (the most recent numbers available) accounted for approximately 64% of the total number of international migrants (International |Labour Organization, 2018) are noticeably absent from these analyses. While the issue of transnational, circulatory, and temporary labor migration has generated considerable interest in the fields of sociology, migration studies, social and cultural anthropology, and human geography, the learning dimensions of migrant laboring have largely been neglected by adult educators and are therefore undertheorized and not well understood (Sawchuk & Kempf, 2008).

In response, this article offers an analysis of learning as it takes place in Canada's Seasonal Agricultural Worker Program (SAWP), a long-standing bilateral transnational circulatory labor management agreement between Canada, Mexico, and several Caribbean countries. Unlike immigrants and refugees to Canada, SAWP workers do not have access to federally funded educational programs for newcomers, such as English as an Additional Language instruction. SAWP workers’ systemic exclusion from state-funded adult education programing could help to explain why their experiences of learning have been overlooked by scholars of adult education. Given this population's lack of access to formal and nonformal learning opportunities, there is a need to examine migrant farm workers’ learning in relation to the informal and experiential social activities in which they participate in their everyday lives (Alenius, 2016; Morrice, 2018).

This article thereby develops an analysis based on findings derived from a research project that explored how the various precarities inherent to transnational labor migration in the context of Canadian agriculture shape the learning experiences of transnational migrant workers. The article engages with the conceptual lens of situated learning (Lave, 1993, 2019; Lave & Packer, 2008; Lave & Wenger, 1991), defined by Lave (1993) as “changing participation in the culturally designed settings of everyday life” (p. 6). This theoretical lens approaches learning as something which is positioned within particular social contexts as constituted by everyday human activity. Simply put, learning is synonymous with ongoing participation in the everyday social world (Lave, 2019). For SAWP workers, who are relatively isolated from the communities where they reside (Horgan & Liinamaa, 2017), the everyday social context is largely limited to the worksite (Perry, 2018). As part of their contractual arrangement, SAWP workers must reside with their co-workers on their employers’ properties in employer-provided dormitories (colloquially known as “bunkhouses”). As a result, their social worlds principally revolve around the relationships they develop with their fellow co-workers (Reber, 2021). In order to tease out the learning implications of transnational labor migration, the article therefore offers an analysis of SAWP workers’ situated participation in workplace-dormitory life.

## Literature Review

The literature review section comprises two subsections. The first subsection reviews the context of migrant agricultural labor in Canada. The second subsection reviews scholarship on situated learning theory.

### Context: Canada's SAWP

The SAWP was introduced in 1966 and the program has since become an established component of Canada's agricultural industry (Preibisch, 2010). The most recent numbers bear this out. In 2018, there were 54,734 migrant farm workers employed in Canada, a number that currently accounts for approximately 30% of the total workforce in Canadian crop production (Statistics Canada, 2019, 2020). SAWP workers are permitted to work in Canada temporarily during the agricultural season. Workers themselves are recruited from rural areas in their home countries and are chosen on the basis of their experience as small-scale farmers. Most workers have low levels of formal education and generally have not completed high school. Typically, workers who decide to work transnationally in Canada do so because they can make more money as migrant worker than they can tending their own fields, and are thus supporting their families and their own farming operations through transnational remittances (Binford, 2013). More than just an avenue for addressing labor shortages, Canada's migrant farm worker programs effectively introduce increased flexibility into the agri-food labor market, providing employers with excessive control over their workforce in order to maximize food production (Preibisch, 2010). This labor control results in an intense competition for jobs, negatively affecting workers’ relationships with each other (Perry, 2018). This is accomplished through legal citizenship restrictions, employers’ control over work permits, social isolation, and restrictive housing regulations.

Unlike federal programs that admit immigrants and refugees to settle in Canada permanently, migrant agricultural workers have no pathway to permanent residence, and must leave Canada at the end of their employment contract, which normally lasts 6–8 months (heretofore referred to as a “season”). Many workers strive to make a good impression on their employer, as they can be to return to the same workplace year after year, often for many years (Binford, 2013). Increasing the power that employers have to develop exploitative workplace practices, federal regulations require that SAWP workers’ work permits be tied to an individual employer, effectively restricting workers’ freedom of movement and employment mobility. SAWP workers are therefore not legally permitted to quit an undesirable job and to find another without risking immediate repatriation (Perry, 2020). Employers in effect have ultimate control over their employees’ ability to legally reside in Canada. As a result, the constant threat of repatriation has evolved as a workplace practice aimed at maximizing worker productivity (Basok et al., 2014), as workers are constantly afraid that any behavior that may be seen as disruptive of workplace norms could result in premature repatriation, not being invited back to the farm, or even blacklisting from the program.

Federal regulations also require that workers do not travel with their families and that they must reside on employer property while they are in Canada. Farm employers are contractually obligated to house their workers in on-site communal dormitories, known colloquially as “bunkhouses.” These dormitories are typically segregated spaces organized by gender (i.e., women only/men only) and nationality (i.e., Mexican only/Jamaican only). This living-at-work requirement increases the level of control that employers have over their employees, thus increasing risks of abuse, exploitation, and health-related injuries (Perry, 2018; Vosko & Spring, 2021). For example, recent research reveals how cramped, overcrowded and ultimately unsafe bunkhouse conditions have greatly magnified workers’ vulnerability to contracting COVID-19 (Bejan et al., 2021; Lavigne, 2021; Vosko & Spring, 2021). Migrant farm workers are thereby geographically estranged from their intimate support networks and are forced to live on the worksite in dangerous and substandard living conditions with their fellow co-workers, in many cases physically separated from surrounding communities (Horgan & Liinamaa, 2017).

In their daily lives, most workers do not have access to organized collective forms of support. With regards to union representation, most provinces in Canada do grant collective bargaining rights to farm workers, which in effect includes SAWP workers. Ontario (the province where this research was conducted, and where most SAWP workers reside) is an exception (Russo, 2018). Even in provinces where SAWP workers can legally engage in collective bargaining, however, workers who do join unions are at risk of being blacklisted from the program by sending state officials. Union engagement, even in jurisdictions where it is legally permitted, therefore contributes to the complex dynamics of SAWP workers’ deportability (Vosko, 2016). There do exist grassroots organizing initiatives, such as Justicia for Migrant Workers (in British Columbia and Ontario) that aim to engage workers in political struggle. Workers who take part in these organizing efforts are also at increased risk of deportation (Paz Ramirez & Chun, 2016).

With a lack of access to collective and even familial support networks, the vast majority of SAWP workers must consequently develop individual strategies for navigating a challenging workplace situation in which both the employer and the state have an inordinate amount of power. On an everyday scale, migrant farm workers must learn to negotiate a complex bunkhouse-workplace nexus in which a multigenerational cohort of workers not only labor together, but also share intimate living spaces, such as communal bedrooms, bathrooms, and kitchen facilities (Perry, 2018). These ultimately strained workplace relationships develop within the broader political economic context whereby the challenges of precarious citizenship status intersect with the financial pressures to send regular remittances to support their families back home (Wells et al., 2014).

### Situated Learning Theory

Much of the scholarship in the area of adult education and immigration is focused on learning related to the settlement and integration experiences of recent immigrants and refugees and examines newcomers’ experiences of learning through state-funded pedagogical interventions, in particular language instruction (Balyasnikova & Gillard, 2021), citizenship learning (Fejes & Dahlstedt, 2020), and employment preparation programs (Maitra, 2017). Given their precarious and temporary citizenship status, SAWP workers have very limited access to formal or nonformal learning opportunities. For several years, Frontier College, a well-known literacy organization in Canada, placed youth volunteers on several farms to provide some access to literacy and EAL instruction for migrant farm workers. While innovative, this small-scale initiative was limited in scope and underfunded. It is no surprise, consequently, that SAWP workers’ learning experiences have been neglected in the adult education literature. This lack of access does not mean, however, that informal learning is not an important element contributing to the labor migration experience. The concept of situated learning offers a distinct window for understanding migrant farm workers’ experiences of learning as it occurs in their everyday lives, how this learning may impact their experiences of migration and work, and how it may play a role in reproducing the social practices associated with Canada's continued engagement with transnational labor migration in agriculture.

Located within a broad conceptualization of experiential learning (Fenwick, 2000), the situated perspective maintains that learning is grounded in the everyday activities and social contexts in which individuals participate. In short: “Learning is situated. There are no exceptions” (Lave, 2019, p. 139) and “to participate in practice *is* to be learning” (Lave & Packer, 2008, p. 40). These are the central ideas that have emerged from Jean Lave's extensive ethnographic investigations of learning as an integral component of social practice. Situated learning is a concept that was first developed by Lave and her colleague Wenger three decades ago (Lave & Wenger, 1991). Drawing from ethnographic analyses of apprenticeship learning from a broad array of contexts, Lave and Wenger argue that “learning” should be thought of as something apart from “education” or “pedagogical strategies.” Rather than thinking about learning as a special kind of activity that is limited to the distinct realm of “schooling,” they assert that learning is a fundamental aspect of people's participation in the lived social world. Thinking about learning as a central feature of social participation shifts attention away from learning as an epistemological activity limited to the realm of “knowledge production” or “skill formation” (Lave & Packer, 2008). Instead, their work facilitates an ontological understanding of learning as “an evolving, continuously renewed set of relations” (Lave & Wenger, 1991, p. 49), and as something which is subsumed within “the everyday production of ongoing practice” (Lave, 2019, p. 130). Questions related to learning thus become questions about how participants change through their evolving participation in social practice, and how social practices are produced and reproduced through people's participation in them (Lave, 2019; Lave & Wenger, 1991).

Seen through this lens, learning is an activity that is deeply implicated in processes of identity and community formation, and the ongoing production and reproduction of organizational and institutional practices. Drawing from ethnographies of apprenticeships, Lave and Wenger's (1991) conceptualization of learning developed from in-depth analyses of apprentices’ relationships with master craftspeople, each of whom were contributing differently to a “textured landscape of participation” (Lave, 2019, p. 135). They demonstrate how learning evolves as a central element of engaging with the conflicts that inevitably arise between masters and apprentices, including in navigating the reality of how apprentices’ sustained participation in the realm of work encompasses the eventual replacement of masters themselves: newcomers eventually become old-timers, who themselves are eventually replaced.

A contradiction thus lies at the heart of situated learning theory. In short, processes of learning are implicated in cycles of social reproduction in such a way that individual social actors are constantly at risk of displacement in order to ensure the ongoing viability of a particular community of practice (Katz, 2004). In response to critiques that the learning implications of this contradiction have not been fully examined, in her more recent work (2008), Lave, drawing from Lefebvre (2004), expands upon how everyday life, the site of learning, can be a place of alienation; a site which is embedded in the uneven rhythms of capital. Given the inordinate power imbalance between employers and employees within Canada's SAWP, these rhythms, which are marked by circulatory transnational migration, the constant threat of deportation, and the need for workers to support the financial sustainability of their families, produce a particularly coercive set of relations (Reid-Musson, 2018), one that permeates the learning experienced through work and migration.

As I shall demonstrate through the data section below, for SAWP workers learning is rooted in the intergenerational conflicts that inevitably shape their experiences of life on Canada's farms (Perry, 2018), and is therefore fraught with struggle. I argue that through navigating these fraught relationships, workers learn to *become* “migrant workers,” and as such, this learning, situated in everyday relations of transnational capital, inadvertently supports the “practical reproduction” (Lave, 2008, p. 42) of Canada's labor migration regime itself.

## Methods

This article is based on data derived from a qualitative study that incorporated semi-structured interviews with SAWP workers in Essex County, Ontario. Known colloquially as the “Greenhouse Capital of North America,” I chose Essex County for this research because it is a major site of agricultural production in Canada. Currently, approximately two-thirds of Ontario's 3000 greenhouse acres are located in the area (Hill, 2020; Ontario Greenhouse|Vegetable Growers, 2021), and the greenhouse industry accounts for approximately one-third of the jobs filled by migrant farm workers in Canada (Statistics Canada, 2020). Because of the large scale of agricultural production in Essex County, the region is a primary destination for migrant farm workers coming to Canada. Leamington, an Essex town where many of the greenhouse operations are located, currently accounts for more than 10% of the total number of migrant farm workers working across the country (Mojtehedzadeh et al., 2017).

The number of migrant workers employed in Canada's agricultural industry increased by 75% in the past decade, growing from 31,200 total workers in 2011 to 54,734 in 2018 (Statistics Canada, 2020; Zhang et al., 2021). While the numbers have grown, the structure of Canada's SAWP has not changed in decades, and the bunkhouse-labor system described in this article has contributed to the heightened vulnerability to workplace injury and death during the coronavirus disease 2019 (COVID-19) pandemic (Bejan et al., 2021). The pandemic has indeed increased the urgency surrounding the need to improve conditions for migrant farm workers in Canada (Office of the Auditor General of |Canada, 2021).

With regard to researcher positionality, I had worked as a migrant farm worker advocate and volunteer outreach worker for Frontier College in Leamington for several years prior to conducting this research. As a part of this volunteer work, for two agricultural seasons I was placed on farms to support small-scale EAL interventions with SAWP workers from Mexico. In this role I lived in employer-provided bunkhouses and worked on farms alongside my SAWP co-workers. During the off-seasons, I traveled with my co-workers to their home countries and met their families. After these two years of working on farms, I spent one year managing a small worker center in Leamington that offered EAL and literacy instruction on weekends, taught by volunteers. While I identify as a white English-speaking Canadian, I learned Spanish during this period and developed a strong network of friends among SAWP workers in the region. I had therefore established a reputation and had built trust among workers in Leamington. My first-hand experience of bunkhouse living provided an important experiential backdrop to this project and helped to break the ice during one-on-one interviews, which were conducted in both Spanish (with Mexican workers) and English (with workers from the Caribbean).

For this project, I conducted semistructured interviews with 25 SAWP workers. I managed the recruitment through my own connections to the migrant worker community. I used snowball recruitment. SAWP workers thus heard about the project through word of mouth and contacted me directly to set up a time to be interviewed. Interviewees included a mix of newcomers and old-timers. The youngest participant was 22 and the oldest was 62, and the number of agricultural seasons worked in Canada ranged from 1 year to 27 years. All participants lived in bunkhouses at their respective places of employment at the time of research.

Given workers’ vulnerability to employer and co-worker reprisals for participating in research, all interviews took place in a private location, such as in my car, or in a local church basement that I had rented for this express purpose. Interviews focused primarily on workers’ first-hand experiences of navigating life in Canada. The flexibility of the semistructured format provided the space for participants to share their experiences and individual viewpoints (Merriam & Tisdell, 2016). All interviewees are provided with a pseudonym.

Participants themselves did not discuss “learning” directly, and so I analyzed the transcripts with a view to noticing patterns that highlight participants’ “changing practice in a complex world” (Lave, 2019, p. 133). Themes thus emerged from participant testimony and were examined through the various conceptual lenses excavated from the extant literature on workplace learning and learning in the context of immigration. It became clear that the learning themes that emerged from the interviews were most often connected to the everyday experience of negotiating intimate relationships at work, and how this negotiation was implicated in participants’ sense of self and their experiences of labor migration. It is through this analysis that the conceptual alignment of situated learning thus evolved. I worked with a native Spanish speaker to translate Spanish quotes into English.

## Presentation of Findings

### “One Learns to Adapt”: Learning to “Become” a Migrant Farm Worker

In many cases, the living-at-work policy described above produces an environment hostile to community formation. Specifically, newcomers discussed entering a situation in which group rituals effectively degrade worker solidarity, while old-timers discussed the need to acclimatize newcomers in order to reduce what they conceived of as behavior that could result in collective harm, such as overtly resisting difficult workplace conditions. In many cases, these everyday social practices resulted in newcomers inevitably learning to adapt to exploitative conditions. No participant expressed this more plainly than Benjamin, a 49-year-old worker from Mexico in his 13th season, who after a long pause replied, stoically: “it's a little bit difficult, but one learns to adapt.” There was a sense from many participants that “learning to adapt” was not only difficult but necessary. In the words of Mauricio, a 26-year-old worker from Mexico in his first season who feared the reprimand of older co-workers: “when workers don’t adapt, there can be trouble.”

But what does “learning to adapt” mean in this context? In his personal reflections on adaptation, Ignacio, a 62-year-old worker from Mexico in his 20th season, referred to the need to “put on a mask” for the eight months that he is in Canada every year. This he described as an outward performance of a public self that is accepting of difficult conditions, regardless of how this performance may contradict his inner sense of self and dignity. The realization that he needed to adapt in order to survive first materialized when, as a novice SAWP worker, he witnessed the deaths of two co-workers as the result of a workplace accident. For Ignacio, this incident triggered a great deal of self-reflection through which he came to recognize the importance of psychologically preparing himself for a life of perpetual circulatory labor migration. He described how he felt his survival in the program required suspending parts of his identity that may interfere with the sacrifice required to be a migrant farm worker in Canada. While in Canada, Ignacio's suspension of these aspects of his identity constituted a personal transformation, however temporary, from Ignacio as husband, father, and friend, into Ignacio as “migrant worker.” Many old-timers described similar approaches to adjusting to life as a migrant farm worker, and described this process as intentionally preparing for pain and mentally accepting the reality that work in Canada will consistently fall short of their expectations.

Many younger workers who witnessed this process among old-timer co-workers actively tried to resist this outward presentation of adapting to poor workplace and immigration conditions, as they interpreted it as simply giving in to exploitation. In the words of Hidalgo, a 26-year-old worker from Mexico in his second season: “the problem is not the employers, but our very own people. We give the power to the employers to do as they like. That's the problem.” Ívan, a 36-year-old worker from Mexico who had worked in Canada for four seasons, described his initial reaction to what he perceived as old-timer complacency thus: “When I came here, and being with eight guys in one room, that thing drive me crazy. I said to myself I’m not gonna be in the program 24 years. I think, ‘I’m not capable of that’.” When he first witnessed his co-workers putting up with conditions that he felt were unacceptable he said: “I was like open eyes when I see a lot of things going on here.” Eventually, his frustration with social isolation, cramped living conditions, and with feeling trapped led to a direct confrontation with his employer, which resulted in his immediate repatriation to Mexico.

For older workers worried about their jobs however, developing a “migrant worker” identity as a means of withstanding the pressures of a transnational labor program that, in the words of John is “a very stressful piece of work,” was described as an essential aspect of surviving life in Canada, and thus of being able to support their families economically. Having to compete with newcomers further complicates this reality. In the words of Ignacio:

If I didn’t need to come to Canada, then I just wouldn’t come, because for me it is very difficult, because of my age. Imagine, I am working with young men who are 25 years old. There is one guy right now that I think is 22. There is no way I am going to beat him at work.

For newcomer workers, this initiation to migrant farm work was very frustrating, and they often found themselves socially isolated when attempting to resist this initiation into the program. All participants described the psychological toll that this struggle between learning to adapt and learning to resist provoked.

In Canada's SAWP, relationships between old-timers and newcomers are fraught with struggle and frustration. Old-timers, many of whom have been going back and forth to work in Canada for ten or more years, have a vested interest in maintaining the social relations that have benefited them financially, regardless of the potential physical and psychological harms. Many newcomers, however, are unconvinced by this strategy, and offer resistance as a means of countering exploitative working and immigration conditions. These are the social relations in which workers must participate, and which ultimately shape workers’ learning. Living at work intensifies these relations. The bunkhouse, more than just a place for workers to lay their heads at night, has become a focal point of social conflict, and thus a site of informal and experiential learning—one that potentially exposes a “dark side” (Morrice, 2013) of labor migration's everyday pedagogies.

### “La Lucha por la Ducha”: Situating Intergenerational Conflict in the Bunkhouse

Given the fraught co-worker relationships that develop within the SAWP, understanding migrant farm workers’ everyday socio-physical environment is a key component for developing a situated learning analysis of agricultural labor migration. To understand workers’ everyday learning, it is important to explore participants’ descriptions of navigating the workplace social relations described above in the context of the SAWP living-at-work arrangement.

Participants described a chaotic work-life environment marked by constant pressure, anxiety, and strained relationships. Referring to the continuous demands to produce in tandem with the ceaseless ambient threat of deportation, Ignacio described a life punctuated by “tremendous pressure, beyond what would be considered normal.” In the words of Enrique, a 40-year-old worker from Mexico in his ninth season, “we each do the work of five Canadians.” Conversations often turned to how the stress of effectively living at work in a space that all participants described as overcrowded and facilitative of dysfunctional relationships. Participants for example discussed how many workers did not have space to unpack their bags, and so lived out of suitcases for the duration of their 8-month contracts. John, a 48-year-old participant from Trinidad & Tobago in his 13th season described an environment full to the brim with hungry and fatigued workers desperate to eat and desperate to rest, but always fighting each other in cramped and inhospitable shared spaces, such as the kitchen and bathroom. This is a dynamic that Mauricio described as “la lucha por la ducha (the struggle to bathe).” Mauricio described how his bunkhouse had an informal hierarchy for the use of amenities such as the shower or kitchen whereby the most senior workers always received preferential access. As a worker in his first season, Mauricio would often end the day without showering or cooking. John, whose bunkhouse had no such rules, described his dormitory dynamic thus:

When I go home, I got to rush to cook, gotta rush to bathe, because you won’t get hot water. You have nine guys and you just have two baths. Some guys wanna wash soiled clothes. So, some guys come home, rush in the kitchen, PA NA NA NA NA [rapid hand gestures and staccato sound effects], you hear pots tumble down … some guys will sit down and say ‘I will cook tonight’ … and they watch the clock, 10, 11 o’clock, them guys not gonna cook. They get up at 5 in the morning and start to prepare self for 6.

In many dormitories, this dynamic produced competitive and even hostile relations among workers. Some of the most emotional testimony came from participants examining the difficult relationships between younger and older workers. Among all participants, there was a general perception that each worker posed a significant risk to the livelihood of the other, leading to resentment and distrust between newcomers and old-timers. On this topic, Jeronimo, a 46-year-old worker from Mexico in his 9th season claimed: “the workers who have a lot of experience don’t always treat new workers well. They think they will take their jobs away.” For their part, many of the old-timers worried that new workers could cause major problems in the dormitories, and discussed how it is the responsibility of old-timers to ensure the maintenance of a “well-organized” (Emilio, 61-year-old worker from Mexico, 27th season) bunkhouse. Old-timer participants discussed the need to prevent novice workers from upsetting the finely tuned dynamics of survival that they felt they had developed over many years, often over decades. For many novice worker participants, this was experienced as old-timers exploiting their experience in an effort to maximize their power in the dormitory and in the workplace, and “this power they use to enact their revenge” (Mauricio) against younger workers. In the words of Hidalgo, a 26-year-old worker from Mexico in his first season: “There is a lot of selfishness. Many times, older workers don’t want to teach what they know.”

For SAWP workers, the problem of learning to adapt to difficult living and working conditions produces a workplace environment that is fraught with conflict between old-timers and newcomers. This conflict is intensified by the geographical convergence of the workplace and living space. What are the learning implications of living and working in this highly emotionally charged environment?

## Discussion

The performance of deferential worker identity and its subsequent dissemination from old-timers to newcomers are crucial elements contributing to farmworkers’ ongoing and changing participation in the everyday cultural dynamics of transnational labor migration. This learning is ultimately implicated in supporting the ongoing viability of Canada's SAWP itself, thereby contributing in part to a cycle of organizational reproduction.

Canada's SAWP produces a highly “textured landscape of participation” (Lave, 2019, p. 135) for workers. The troubled relationships described in the findings evolve from an environment designed to maximize food production via policies that augment migrant labor flexibility and which boost employer control (Preibisch, 2010). Practices, such as the ongoing threat of deportation (Basok et al., 2014), and policies, such as the requirement that workers be housed on employer property (Perry, 2018), are deeply implicated in the everyday production of worker relations (Lave, 2019). If we consider learning as an essential component of people's active participation in the social world, then the question inevitably arises as to what these workers’ experiences of negotiating intergenerational co-worker relationships can tell us about learning in this context, however fraught this learning experience may be. By applying the concept of situated learning to these exceptionally “alienated, commoditized relations,” the findings highlight the “dark side” of everyday learning, an area of research that Jean Lave herself and others have argued requires further illumination (Fuller et al., 2005; Lave, 2019, p. 140). Examining the daily interactions that occur in this distinctive workplace ecosystem, I argue that learning takes place as a part of workers’ ongoing struggles for sparse resources and is thus an important and underappreciated aspect of migrant workers’ experiences on Canada's farms.

In an effort to maximize their survival in the program, over time, workers learn to perform the social behaviors that are expected by their employer and by wider societal norms (Goffman, 1959). This is a process that Ignacio above refers to as “putting on a mask”—the formation of an outward performance that reaffirms the discipline of coercive immigration and employment controls, controls that, through the mechanism of living at work, are “enforced at the level of everyday social life” (Choudry & Smith, 2016, p. 12). The project findings suggest that this performance is an important means through which migrant worker identities are expressed and communicated among workers. Eventually, through negotiating intimate living-at-work relationships, particularly those between old-timers and newcomers, workers learn to develop a collective capacity to outwardly perform the gestures associated with deferential subjectivity. Old-timers attempt to “teach” this identity through bunkhouse rules and behaviors intended to acclimatize newcomers to challenging conditions. While the novice workers interviewed for this study were resistant to these dynamics, eventually, in the words of Benjamin, “one learns to adapt.” It is through the negotiation of these relations that “practices, participants, and ways of participating change” (Lave, 2019, p. 130), thus highlighting how the socio-experiential learning associated with life as a migrant farm worker in Canada is implicated in migrant identity formation as a means of sustaining the long-term economic survival of migrant workers and their families.

SAWP workers’ changing participation in the social dynamics that permeate daily life in the program further underscores the paradox unearthed by situated learning theory whereby processes of learning are intimately connected to cycles of organizational social reproduction (Katz, 2004; Lave, 2019). Based on Lave and Wenger's (1991) analysis of apprenticeship ethnographies, eventual participant displacement can be understood as an important element to the long-term viability of an organization. In the case of Canada's SAWP, the risk of displacement is compounded by workers’ deportability and by the constant threat to one's livelihood that this deportability represents (Basok et al., 2014). While the program's ongoing sustainability is ensured through the uneven politics of global transnational capitalism (Preibisch, 2012), the learning described above may also be implicated in the reproduction of a reliable transnational workforce from the ground up. Workers’ everyday experiential learning may thus contribute to the ongoing reproduction of the system itself. This is evident from the high rates of return of old-timer research participants (14 participants had been returning to Canada for over 10 consecutive years).

High rates of return support the ongoing success of Canada's SAWP, and the socio-experiential process of learning to adapt is a key component of worker longevity. For old-timers, the outward performance of a deferent self-offers a competitive advantage over novice workers, workers with whom they may not otherwise be able to compete in terms of productivity. Indeed, Ignacio attributes his decision to “wear a mask” to his ability to return to Canada every season for the past 20 years. While learning to “become” a migrant worker early in one's tenure can increase the life-long earning potential of SAWP workers, it comes at a high price—including a lifetime away from family and continuous exposure to precarious and exploitative workplace and immigration conditions. This is the “dark side” of situated learning that SAWP social dynamics expose. Newcomers learn to survive life in the program. In so doing, this socio-experiential learning ultimately plays a role in reproducing the coercive social relations that prop up an employment regime that relies on workers from the Global South who are willing to fill temporary work contracts year in and year out for many years. In the words of Mauricio, who was reflecting on his own learning on the farm: “The older workers can really screw you over, but in two years I will be the one able to make life hell.” While he went on to say that he should not be thinking like this, over the course of his first year in the program, he had become resigned to what he perceived as his inevitable future.

## Conclusion

Exposing the learning dimensions of everyday life as a migrant farm worker in Canada reveals a “dark side” to situated learning, one in which processes of learning collide with exploitative employment and immigration practices to generate subjectivities conducive to the production and reproduction of agricultural production and capital. Participant accounts suggest that the SAWP workplace-living environment is one that belies the possibility of building a community of practice that fosters solidarity and trust. On the contrary, this environment engenders coercive everyday social relations that pit migrant workers against each other in a competition for highly valued Canadian jobs. This competition acutely affects the relationships between old-timers and newcomers. Survival strategies aimed at adapting to, as opposed to resisting, exploitative employment and immigration conditions, are circulated from senior workers to novice workers. Through old-timers’ modeling the performance of a “mask” intended to ensure workers’ long-term survival in the program, newcomers become socialized to accept a migrant worker identity that conforms to societal norms. As such, everyday learning in this context involves the process of the ongoing renewal of a coercive set of relations (Lave & Wenger, 1991) on Canadian farms, thereby demonstrating how informal and experiential learning in this context can effectively support the “practical reproduction” (Lave, 2008, p. 42) of Canada's transnational agricultural migrant labor system.
